# Impact of *IFNL4* Genetic Variants on Sustained Virologic Response and Viremia in Hepatitis C Virus Genotype 3 Patients

**DOI:** 10.1089/jir.2019.0013

**Published:** 2019-09-27

**Authors:** Vincent Pedergnana, William L. Irving, Eleanor Barnes, John McLauchlan, Chris C.A. Spencer

**Affiliations:** ^1^ Wellcome Centre Human Genetics, University of Oxford, Oxford, United Kingdom.; ^2^ Laboratoire MIVEGEC (UMR CNRS 5290, UR IRD 224, UM), Montpellier, France.; ^3^ National Institute for Health Research (NIHR) Nottingham Biomedical Research Centre, Nottingham University Hospitals NHS Trust and University of Nottingham, Nottingham, United Kingdom.; ^4^ Nuffield Department of Medicine and the Oxford NIHR BRC, University of Oxford, Oxford, United Kingdom.; ^5^ Centre for Virus Research, MRC-University of Glasgow, Glasgow, United Kingdom.

**Keywords:** hepatitis C virus, direct-acting antivirals, genome-wide association study, candidate genes, *IFNL4*

## Abstract

Hepatitis C virus (HCV) genotype 3 is very prevalent in Europe and Asia and is associated with worst outcomes than other genotypes. Genetic factors have been associated with HCV infection; however, no extensive genome-wide study has been performed among HCV genotype 3 patients. In this study, using a large cohort of 1,759 patients infected with HCV genotype 3, we explore the role of genetic variants on the response to interferon (IFN) and direct-acting antiviral (DAA) regimens and viremia in a combined candidate gene and genome-wide analysis. We show that genetic variants within the IFN lambda 4 (*IFNL4*) locus are the major factors associated with the studied traits, accordingly with observations in other HCV genotypes and with comparable effect sizes. In particular, the functional dinucleotide polymorphism rs368234815 was associated with IFN-based sustained virologic response (SVR) [odds ratio (OR) = 1.5, *P* = 2.3 × 10^−7^], viremia (beta = −0.23, *P* = 8.8 × 10^−10^), and also DAA-based SVR (OR = 1.7; *P* = 4.2 × 10^−4^). Our results provide evidence for a role of genetic variants on HCV viremia and SVR, notably DAA-based, in patients infected with HCV genotype 3.

## Introduction

Chronic hepatitis C (CHC) infection affects more than 71 million people worldwide (WHO 2017). Hepatitis C infection is a major Public Health problem, as CHC patients are at risk to develop severe liver disease such as cirrhosis and hepatocellular carcinoma (HCC), the second cause of death by cancer in the world. Hepatitis C virus (HCV) is a very genetically diverse virus, with 7 genotypes and 67 subgenotypes (Simmonds, 2004). The most frequent genotypes are the genotypes 1 and 3 (Messina and others 2014).

Until recently, the treatment for hepatitis C was based on pegylated interferon alpha (peg-IFN-α) injections in combination with Ribavirin over a period of 24–48 weeks depending on the HCV genotype being treated (Friedman and Contente, 2010). Genotypes 1 and 4 historically showed lower sustained virologic response (SVR) rates than genotypes 2 and 3. The development of direct-acting antiviral (DAA) drugs such as Sofosbuvir has tremendously increased SVR rates in all HCV genotypes (Li and De Clercq, 2017).

Many genome-wide association studies (GWASs) and candidate gene studies have reported strong associations between human single nucleotide polymorphisms (SNPs) and different HCV outcomes such as spontaneous clearance of the virus, response to IFN-based treatment, and progression to liver diseases (Ge and others 2009; Suppiah and others 2009; Tanaka and others 2009; Thomas and others 2009; Rauch and others 2010; Patin and others 2012; Noureddin and others 2013; Aoki and others 2015). The most striking result to date remains the association between SNPs in the IFN lambda (IFNL) 3 and *IFNL4* locus and the clearance of the virus (Ge and others 2009; Tanaka and others 2009; Thomas and others 2009; Rauch and others 2010; Pedergnana and others 2012). However, most of these observations have been made in cohorts of patients infected by HCV genotype 1, the most common genotype in North America and Europe (Ge and others 2009; Thomas and others 2009; Rauch and others 2010). For instance, a functional dinucleotide polymorphism located within the *IFNL4* gene (rs368234815 ΔG/TT, *IFNL4-ΔG* allele creates an open reading frame that allows production of IFN-λ4 protein that is not produced in individuals homozygous for the *IFNL4-TT* allele) has been reported to be associated with viral decline, spontaneous clearance, IFN based therapy, viral diversity, and very recently DAA based therapy in patients infected with HCV genotype 1 (Bochud and others 2012; Prokunina-Olsson and others 2013; Ansari and others 2017; Backus and others 2018).

Host genetic associations with HCV genotype 3 outcomes have not been described extensively, even though this genotype is highly prevalent in Asia and Europe (Messina and others 2014). Moreover, no GWASs have been performed in patients infected specifically with HCV genotype 3. In the context of DAA treatment, where HCV genotype 3 is one of the more complicated genotypes to treat and with uncertainty about the progression of liver disease for patients with SVR, it remains of major importance to further explore the genetic determinants of HCV infection (Foster and others 2015).

In a combined cohort of 1,759 patients infected by HCV genotype 3, we performed an epidemiological study of demographic and clinical variables for 3 different traits related to HCV infection, ie, pretreatment viral load (PTVL), response to IFN therapy, and response to DAA therapy. We describe the evidence for association at 38 previously reported genetic variants located at 33 loci, and in particular within the *IFNL3-IFNL4* locus. Finally, we performed a GWAS of these 3 different traits.

## Materials and Methods

### Patient subjects

The samples used for this analysis were derived from 2 cohorts of patients chronically infected with HCV genotype 3. The first cohort included patients from the BOSON phase 3 randomized open-label trial (Foster and others 2015). The second cohort included British patients recruited by the HCV Research UK (HCVRUK) clinical database and biobank, which includes over 10,000 patients from across the United Kingdom, who have attended a specialist HCV clinic for care/management of their HCV infection (Mclauchlan and others 2017). In these 2 cohorts, we retained only patients that were infected by HCV genotype 3 and for whom sufficient DNA material was available for genotyping. In total, 1,759 patients (486 from the BOSON cohort and 1,273 from the HCVRUK cohort) were eligible for analyses. Due to the multicenter basis of these cohorts, all clinical data included in this analysis were not available in all patients, resulting in different sample sizes for the different HCV outcomes studied (1,498 patients had PTVL data available, 1,258 have had IFN-based therapy, and 409 DAA-based therapy). Some patients were used for 1 trait (PTVL, IFN-based therapy outcome, or DAA-based therapy outcome), while others were used for 2 or 3 traits. The sampling of all the cohorts was approved by the appropriate institutional review boards, and written informed consent was obtained from all patients (Ansari and others 2017; Mclauchlan and others 2017).

### Genotyping and imputation procedures

Genotyping was performed using Affymetrix UK Biobank arrays, which provided for ∼800,000 SNPs. Quality control filtering of SNPs resulted in a total of 387,664 high-quality SNPs (considered as *PolyHighRes* by the SNPolisher software) having a call rate of 95%, and a minor allele frequency (MAF) >5%. Genotype phasing was performed using SHAPEIT2 (Delaneau and others 2012, 2013) and genotype imputation using IMPUTE2 (Howie and others 2009) with standard parameters, using the 1000 Genomes Phase III panel as reference (Abecasis and others 2012). SNPs with an imputation score (information) greater than 0.5 were selected for analysis, resulting in ∼18 million of high-quality imputed SNPs.

### Statistical analyses

Epidemiological analysis was performed using logistic (for IFNRB_SVR and DAA_SVR) or linear regression [for log_10_(PTVL)] as implemented in R [version 3.2.4 (2016-03-10)]. To test for association between human SNPs and HCV outcomes, we performed logistic regression for binary variables or linear regression for continuous variables using an additive model in SNPTEST, adjusted for age, sex, and population structure [10 first principal components (PCs) as assessed using FlashPCA] (Abraham and Inouye, 2014).

## Results

### Epidemiological study

In total, the combined cohort consisted of 1,759 patients, 1,273 from the HCVRUK cohort and 486 from the BOSON cohort; 1,171 (66.6%) patients were males, 584 (33.2%) were female, and 4 had undefined reported sex. For these 5 patients, we used the inferred sex based on the genotypes. The mean age was 51.7 years with no difference (*P* = 0.64) between females and males (51.6 and 51.8 years, respectively). The majority of the cohort (*n* = 1,006; 57.2%) consisted of patients of self-reported white ancestry. The second most prevalent group was patients of self-reported Asian ancestry (*n* = 318, 18.1%). As self-reported ancestry was not available for 435 patients, we used PC analysis of the genetic data to take into account population stratification in the further statistical analysis.

We studied 3 different traits relating to HCV infection (Table 1): (1) viral load measured before the treatment was administered, we used the normalized log_10_ value [log_10_(PTVL)]; (2) the response to peg-IFN-α and Ribavirin treatment (IFNRB_SVR); and (3) the response to DAA treatment (DAA_SVR). The BOSON cohort consisted only of treatment naive patients or patients who failed peg-IFN-α and Ribavirin treatment and was subsequently treated with Sofosbuvir and Ribavirin with or without peg-IFN-α. Patients in the HCVRUK cohort received different regimens of DAAs, including Sofosbuvir alone (*n* = 41, 48.2%) or in combination with Daclatasvir (*n* = 29, 34.1%) or Ledipasvir (*n* = 15, 17.7%). In the following analysis, we decided to combine all different regimens, including Sofosbuvir, with different combinations as “DAA regimen” to ensure sufficient sample size for analysis.

**Table 1.** Outcome Repartition in the 2 Different Cohorts

*Outcome*	*BOSON*	*HCVRUK*	*Total,* n
IFNRB_SVR, *n* (%)			
Responder	0 (0)	717 (100)	717
Nonresponder	238 (44)	303 (56)	541
DAA_SVR, *n* (%)			
Responder	250 (77)	75 (23)	325
Nonresponder	74 (88)	10 (12)	84
log_10_(PTVL)	6.26 ± 0.03	5.69 ± 0.02	5.87 ± 0.02

For the log_10_(PTVL), the mean and the standard error of the mean are reported, in IU/mL.

DAA, direct-acting antiviral; PTVL, pretreatment viral load; SVR, sustained virologic response.

**FIG. 1.**
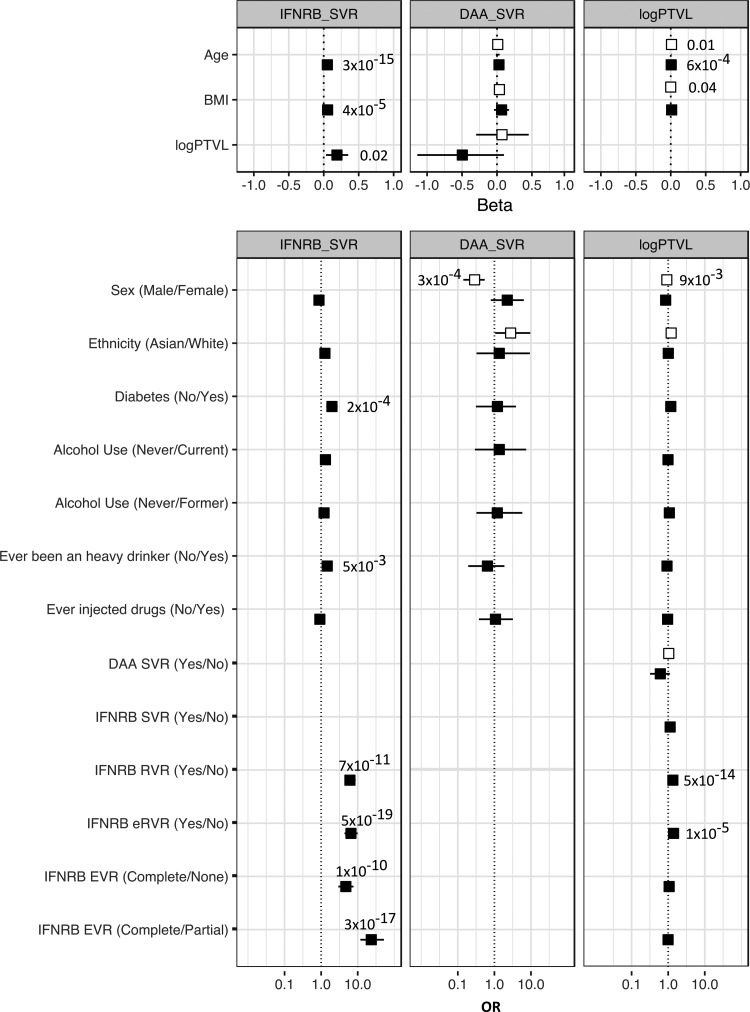
Forest plot of the demographic and clinical characteristics of the patients by HCV related phenotype—response to peg-IFN-α and Ribavirin treatment (IFNRB_SVR) or to DAA treatment (DAA_SVR) and viral load [log_10_(PTVL)]. *Top panel*: linear regression for continuous variables. *Bottom panel*: logistic regression for binary variables. Effect size is given as beta (and 95% CI) for continuous variables and OR and 95% CI for binary variables. *Filled squares* represent the HCV Research UK cohort and *unfilled squares* the BOSON cohort. *P* values are indicated when significant in a univariate test (<0.05). CI, confidence interval; eRVR, extended RVR; EVR, early virologic response; HCV, hepatitis C virus; OR, odd ratio; peg-IFN-α, pegylated interferon alpha; PTVL, pretreatment viral load; RVR, rapid virologic response; SVR, sustained virologic response.

Several clinical factors were available in this cohort, and we performed a regression analysis to assess the association between these clinical factors and the 3 HCV traits of interest (Fig. 1). In these cohorts of HCV genotype 3 infected patients, the risk factors for treatment response previously reported in HCV genotype 1 infection (Lee and others 2014) were also found to be associated with HCV genotype 3 infection. Age, sex, and body mass index (BMI) were consistently associated with SVR and log_10_(PTVL). Alcohol consumption and diabetes, most likely type 2 diabetes even if no specific categorization was available, were associated with a worst response to peg-IFN-α treatment.

### Candidate SNP analysis

**FIG. 2.**
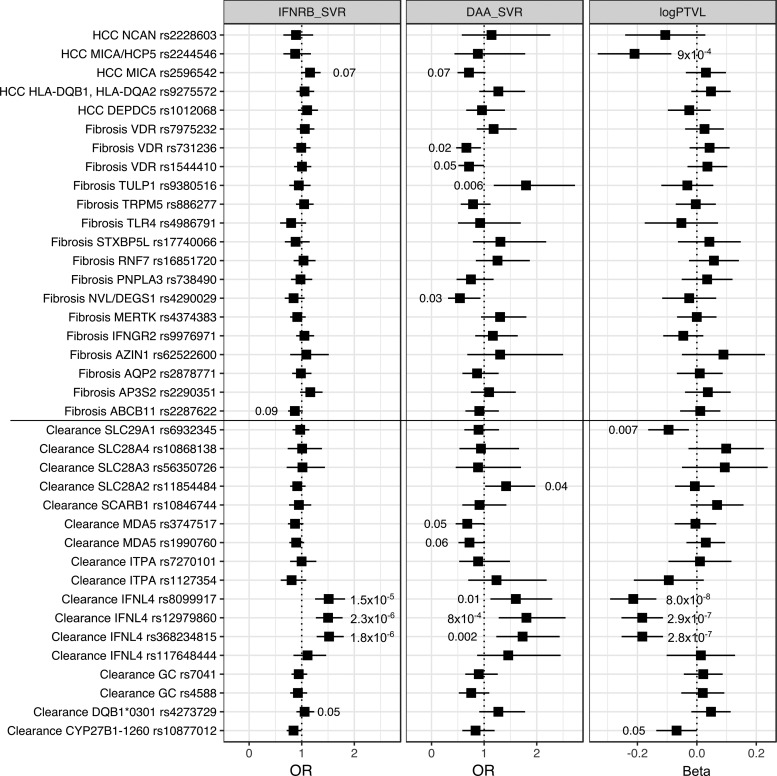
Forest plot of the 38 single nucleotide polymorphisms previously reported by HCV related phenotype—response to peg-IFN-α and Ribavirin treatment (IFNRB_SVR) or to DAA treatment (DAA_SVR) and viral load [log_10_(PTVL)]. Effect size is given as OR and 95% CI. *P* values are indicated when significant in a univariate test (<0.05). DAA, direct-acting antiviral.

Many genetic variants have been associated with either SVR or progression of liver disease (Rau and others 2012). We performed a nonexhaustive review of the literature, as well as a discussion with experts in the field, and selected 38 genetic variants located in 33 loci that have already been reported to be significantly associated with viral clearance (*n* = 17), fibrosis and/or cirrhosis (*n* = 16), or HCC (*n* = 5) (Supplementary Table S1). We used a logistic regression for the 2 binary traits (IFNRB_SVR and DAA_SVR) and a linear regression for log_10_(PTVL), including sex and age as covariates, as well as the cohort of origin, and the plate on which the genotyping was done to account for cohort and plate effect and the 10 first PCs to account for population stratification. Results of the candidate SNP analysis are presented in Figure 2.

In our cohort of HCV genotype 3 infected patients, only 4 out of 17 genetic variants reported in the literature to be associated with SVR were replicated when testing for the association with IFN-induced SVR (*P* < 0.05, no correction for multiple testing). Three were located in the *IFNL4* loci, the other one in the HLA-DQB1*03:01 gene [previously reported to be associated with spontaneous clearance; Duggal and others (2013)].

Only one gene showed consistent association across the 3 traits, the *IFNL4* gene, with 3 of the 4 tested genetic variants significantly associated (rs12979860, rs8099917, rs368234815). The strongest association was found for the SNP rs12979860, which has been previously reported to be associated with clearance of the virus, either spontaneously or after peg-IFN-α and Ribavirin treatment (Thomas and others 2009). Interestingly, in this large cohort of 409 patients treated with DAAs, we observe a strong association between DAA-induced SVR and the SNP rs12979860 of the same magnitude as the association observed with IFN-induced SVR [odds ratio (OR) = 1.5]. The fourth SNP that was tested (SNP rs117648444 [G>A]) is located in an exon of *IFNL4* and substitutes a proline for a serine at position 70 (P70 and S70, respectively). It has been shown to reduce antiviral activity in HCV infected patients (Terczyńska-Dyla and others 2014) to be a confounder of IFN-induced SVR in HCV genotype 3 patients (Bhushan and others 2017).

**FIG. 3.**
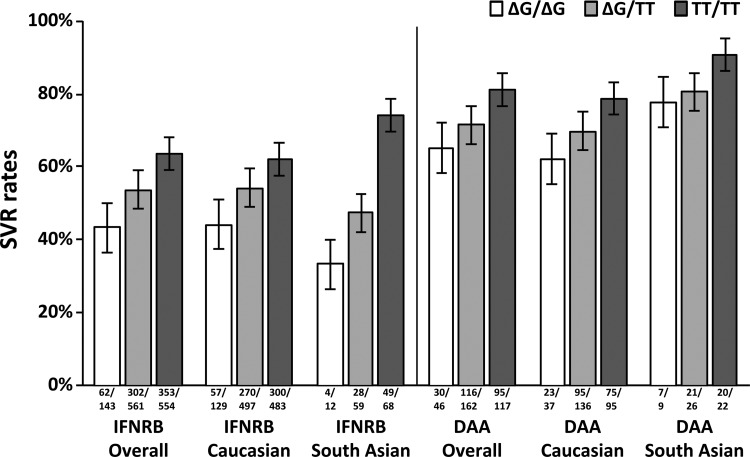
Sustained virologic response rates of HCV genotype 3 patients receiving peg-IFN-α and Ribavirin (IFNRB) or DAA treatment by ethnic origin and *IFNL4* rs368234815 genotypes. IFNRB_SVR (ΔG/ΔG versus ΔG/TT, or ΔG/TT versus TT/TT OR = 1.5, *P* = 2.3 × 10^−7^), DAA_SVR (ΔG/ΔG versus ΔG/TT, or ΔG/TT versus TT/TT OR = 1.7; *P* = 4.2 × 10^−4^), and log_10_(PTVL) (ΔG/ΔG versus ΔG/TT, or ΔG/TT versus TT/TT beta = −0.23, *P* = 8.8 × 10^−10^). IFNL, interferon lambda.

To further explore the *IFNL4* associations with the HCV related phenotypes and in particular with SVR rates, we used imputation to infer SNPs in the *IFNL* locus (see Materials and Methods section). In total, 114 SNPs were imputed with high quality (information >0.5; average maximum posterior call >0.9; MAF >0.05) in this region. Association tests confirmed that the signal was located in the *IFNL3-IFNL4* locus (Supplementary Fig. S1 and Supplementary Tables S2, S3, S4), with all 3 phenotypes showing evidence for association with a set of SNPs in high linkage disequilibrium (*r*^2^ > 0.9) in the region. In particular, rs368234815 showed strong signals for association for IFNRB_SVR (ΔG/ΔG versus ΔG/TT, or ΔG/TT versus TT/TT OR = 1.5, *P* = 2.3 × 10^−7^), DAA_SVR (ΔG/ΔG versus ΔG/TT, or ΔG/TT versus TT/TT OR = 1.7, *P* = 4.2 × 10^−4^), and log_10_(PTVL) (ΔG/ΔG versus ΔG/TT, or ΔG/TT versus TT/TT beta = −0.23, *P* = 8.8 × 10^−10^) (Fig. 3)

**FIG. 4.**
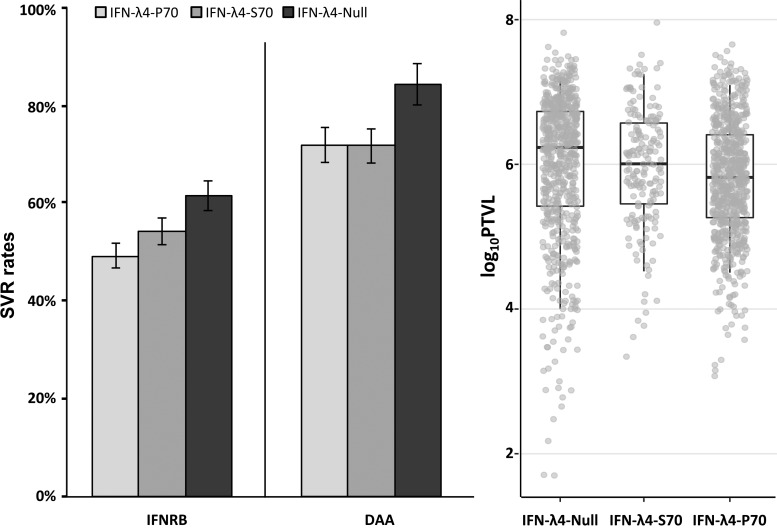
Sustained virologic response rates of HCV genotype 3 patients receiving peg-IFN-α and Ribavirin (IFNRB) or DAA treatment and viral load [log_10_(PTVL)] by IFN-λ4 variants (IFN-λ4-Null, IFN-λ4-S70, IFN-λ4-p70). IFNRB_SVR (IFN-λ4-Null versus IFN-λ4-S70, *P* > 0.1; IFN-λ4-Null versus IFN-λ4-P70, *P* = 2.2 × 10^−5^; IFN-λ4-S70 versus IFN-λ4-P70, *P* > 0.27), DAA_SVR (IFN-λ4-Null versus IFN-λ4-S70, *P* = 0.04; IFN-λ4-Null versus IFN-λ4-P70, *P* = 2.7 × 10^−3^; IFN-λ4-S70 versus IFN-λ4-P70, *P* > 0.97), and log_10_(PTVL) (IFN-λ4-Null versus IFN-λ4-S70, *P* > 0.7; IFN-λ4-Null versus IFN-λ4-P70, *P* = 7.4 × 10^−8^; IFN-λ4-S70 versus IFN-λ4-P70, *P* = 5 × 10^−4^).

We then further investigated the role of the SNP rs117648444. By phasing *IFNL4* SNPs rs368234815 and rs117648444 we observed 3 haplotypes: TT/G (IFN-λ4-Null), ΔG/G (IFN-λ4-P70), and ΔG/A (IFN-λ4-S70). Patients were classified into 3 groups according to their predicted ability to produce IFN-λ4 protein: no IFN-λ4 (2 allelic copies of IFN-λ4-Null, *n* = 771), IFN-λ4-S70 (2 copies of IFN-λ4-S70 or 1 copy of IFN-λ4-S70 and 1 copy of IFN-λ4-Null, *n* = 199), and IFN-λ4-P70 (at least 1 copy of IFN-λ4-P70, *n* = 789). We investigated the effect of the different IFN-λ4 haplotypes on peg-IFN-α and Ribavirin-induced SVR (IFNRB_SVR), DAA-induced SVR (DAA_SVR), and viral load. Compared to IFN-λ4-Null, IFN-λ4-S70 showed no association with IFNRB_SVR (*P* > 0.1) or viral load (*P* > 0.7). IFN-λ4-S70 was marginally associated with DAA-SVR [*P* = 0.04, OR = 2.33, 95% confidence interval (CI): 1.02 to 5.16]. However, IFN-λ4-P70 was strongly associated with IFNRB_SVR and viral load (*P* = 2.2 × 10^−5^, OR = 1.68, 95% CI: 1.32 to 2.13; *P* = 7.4 × 10^−8^, beta = −0.27, 95% CI: −0.17 to 0.37, respectively) and to a lesser extent with DAA_SVR (*P* = 2.7 × 10^−3^, OR = 2.31, 95% CI: 1.36 to 4.06) (Fig. 4).

### Genome wide association study

Taking advantage of the whole dataset of genotypes enriched with imputed SNPs (over 18 million SNPs in total), we performed 3 GWASs for the 3 HCV related phenotypes in our cohort [IFN_SVR, DAA_SVR, and log_10_(PTVL)]. We found a significant association between SNPs in the *IFNL4* locus and log_10_(PTVL) [strongest association signal for rs4803221 (CC vs CG or CG vs GG), beta = −0.28 ± 0.04, *P* = 1.7 × 10^−11^] (Supplementary Fig. S2 and Supplementary Table S5). However, no GWAS significant association (*P* < 5 × 10^−8^) was found for the 2 remaining phenotypes (Supplementary Figs. 3 and 4and Supplementary Tables S6 and S7).

## Discussion

In this study, we performed a genetic analysis of CHC in a combined cohort of 1,759 patients recruited from routine care in a hospital in the United Kingdom and a clinical trial. The major challenge of this study was the collection of clinical data from medical records for patients that were treated for CHC in hospital and not recruited through a standardized protocol. We believe that with the constant falling price of genotyping and sequencing technologies, genome-wide genetic data will become more routinely available for individual within health care systems. Access to well curated clinical dataset becomes a relatively more challenging part of any analysis to understand the genetic basis of health related traits. We explore here the feasibility of this approach in studying 3 phenotypes related to HCV infection.

In these cohorts of HCV genotype 3 infected patients, we explored the association between PTVL and response to IFN or DAA treatment with a range of demographic and clinical variables. The risk factors for HCV outcomes previously reported in HCV genotype 1 infection were also found to be associated with HCV genotype 3 infection. Indeed, age, sex, and BMI were consistently associated with SVR and log_10_(PTVL). Alcohol consumption and diabetes were associated with a worst response to peg-IFN-α treatment. These results confirm that HCV genotype 3 infection has a similar impact to HCV genotype 1 infection, at least in Caucasian and South Asian patients living in the United Kingdom. It also suggests that the differences observed in the SVR rates between HCV genotype 3 and other genotypes must be dependent not only on clinical or demographic features but also most likely on host and/or viral genetic factors.

We then explored the host genetic basis of HCV genotype 3 infection by testing the association between 3 HCV-related phenotypes and a set of 38 genetic variants previously reported to have an impact on HCV infection. The only consistent association across the 3 phenotypes was for genetic variants located within the *IFNL4* locus, confirming the major effect of this gene on HCV related phenotypes in HCV genotype 3 infection. Other signals failed to be replicated, possibly due to a lack of power or a specific effect of the HCV genotype 3 infection.

Several genetic variants (5/21) reported to be associated with fibrosis or HCC showed significant association with DAA-induced SVR in our analysis. This is likely due to the specific nature of the patients treated with DAAs. Indeed, in the early phase of DAA treatment, patients treated were the patients with the most advanced liver disease. Our cohort of DAA-treated patients is thus enriched in patients with fibrosis or cirrhosis.

We also report a strong association signal between rs368234815 genotypes and DAA response. This observation confirms the recent report of Backus and others in a much larger cohort and in a different HCV genotype. It suggests that *IFNL4-*Δ*G* allele is also a strong predictor of SVR when using DAA regimens and that these IFN-free regimens are still susceptible to patients' intrinsic *IFNL4* expression.

By phasing the *IFNL4* SNPs rs368234815 and rs117648444, we assessed the role of the exonic P70S variant on SVR and viral load. We observed that the patients producing the IFN-λ4-S70 protein were not significantly different from the patients who do not produce IFN-λ4 protein in terms of IFN-induced SVR rate and viral load. In comparison, the IFN-λ4-P70 variant was strongly associated with a lower rate of IFN-induced SVR and lower viral load. These results are consistent with the reduced antiviral activity of the IFN-λ4-S70 variant and the higher IFN-based SVR rate in HCV genotype 3 patients compared to the IFN-λ4-P70 variant previously reported. This confirms the protective role against HCV of the low-activity IFN-λ4-S70 compared to the high-activity IFN-λ4-P70 variant. The effect of the IFN-λ4-S70 variant on DAA-SVR was similar to the IFN-λ4-P70 variant, but the observed association (*P* = 0.04) was marginal and will need to be replicated in a bigger sample.

We finally performed GWASs for the 3 phenotypes. No genome-wide significant signal was observed for IFN and DAA based treatment, most likely due to a lack of power in this small sample, as suggested by the QQ plots (Supplementary Figs. S2, S3, S4). GWASs for PTVL showed a significant signal within the *IFNL4* locus (*P* = 1.7 × 10^−11^). This observation highlights the interest of using a continuous biological variable as a proxy phenotype for a disease phenotype when exploring the genetic basis of a disease. Indeed, sample size was similar between IFN-based treatment and viral load, but significance level was only reached for viral load GWASs.

In summary, we report the first large-scale genetic study of HCV genotype 3 infections. We confirmed previously reported observation in other HCV genotypes and the importance of *IFNL4* gene in the biology of HCV infection.

## Supplementary Material

Supplemental data

Supplemental data

Supplemental data

Supplemental data

Supplemental data

Supplemental data

Supplemental data

Supplemental data
