# Supplementary material for: Impact of IFNL4 Genetic Variants on Sustained Virologic Response and Viremia in Hepatitis C Virus Genotype 3 Patients
Source: J Interferon Cytokine Res. 2019 Sep 27;39(10):642–9. doi: 10.1089/jir.2019.0013 (PMC6767867; doi:10.1089/jir.2019.0013)
Supplement: Supplemental data [file Supp_Table1.pdf]

Supplementary Table S1. Genetic Variants Associated with Hepatocellular Carcinoma, Liver Disease (Fibrosis), and Treatment Outcome

Phenotype	*SNP*	*CHR*	*Gene*	*Reference (DOI)*
HCC	rs2228603	19	*NCAN*	10.1016/j.jhep.2014.06.006
HCC	rs2596542	6	*MICA*	10.1038/ng.809
HCC	rs9275572	6	*HLA-DQB1, HLA-DQA2*	10.1038/ng.809
HCC	rs2244546	6	*HCP5/MICA*	10.1016/j.jhep.2013.04.032
HCC	rs1012068	22	*DEPDC5*	10.1038/ng.876
Fibrosis	rs7975232	12	*VDR*	10.3851/IMP2018
Fibrosis	rs731236	12	*VDR*	10.3851/IMP2018
Fibrosis	rs1544410	12	*VDR*	10.3851/IMP2018
Fibrosis	rs9380516	6	*TULP1*	10.1053/j.gastro.2012.07.097
Fibrosis	rs886277	11	*TRPM5*	10.1097/FPC.0b013e32834c3e74
Fibrosis	rs4986791	9	*TLR4*	10.1097/FPC.0b013e32834c3e74
Fibrosis	rs17740066	3	*STXBP5L*	10.1097/FPC.0b013e32834c3e74
Fibrosis	rs16851720	3	*RNF7*	10.1053/j.gastro.2012.07.097
Fibrosis	rs738490	22	*PNPLA3*	10.1002/hep.24350
Fibrosis	rs4290029	1	*NVL/DEGS1*	10.1097/FPC.0b013e32834c3e74
Fibrosis	rs4374383	2	*MERTK*	10.1053/j.gastro.2012.07.097
Fibrosis	rs9976971	21	*IFNGR2*	10.1136/gut.2009.202267
Fibrosis	rs62522600	8	*AZIN1*	10.1097/FPC.0b013e32834c3e74
Fibrosis	rs2878771	12	*AQP2*	10.1097/FPC.0b013e32834c3e74
Fibrosis	rs2290351	15	*AP3S2*	10.1097/FPC.0b013e32834c3e74
Fibrosis	rs2287622	2	*ABCB11*	10.1016/j.jmoldx.2017.07.005
Clearance	rs6932345	6	*SLC29A1*	10.1111/j.1478-3231.2011.02727.x
Clearance	rs10868138	9	*SLC28A4*	10.1016/j.jhep.2012.11.027
Clearance	rs56350726	9	*SLC28A3*	10.1016/j.jhep.2012.11.027
Clearance	rs11854484	15	*SLC28A2*	10.1016/j.jhep.2012.11.027
Clearance	rs10846744	12	*SCARB1*	10.1038/srep32303
Clearance	rs3747517	2	*MDA5*	10.1002/hep.27951
Clearance	rs1990760	2	*MDA5*	10.1002/hep.27951
Clearance	rs7270101	20	*ITPA*	10.1053/j.gastro.2010.06.071
Clearance	rs1127354	20	*ITPA*	10.1053/j.gastro.2010.06.071
Clearance	rs8099917	19	*IFNL4*	10.1038/ng.449
Clearance	rs12979860	19	*IFNL4*	10.1038/nature08463
Clearance	rs368234815	19	*IFNL4*	10.1038/ng.2521
Clearance	rs117648444	19	*IFNL4*	10.1016/j.jcv.2014.01.006
Clearance	rs7041	4	*GC*	10.1002/hep.25848
Clearance	rs4588	4	*GC*	10.1002/hep.25848
Clearance	rs4273729	6	*DQB1*0301*	10.7326/0003-4819-158-4-201302190-00003
Clearance	rs10877012	12	*CYP27B1-1260*	10.1016/j.jhep.2010.08.036

HCC, hepatocellular carcinoma; SNP, single nucleotide polymorphism.
