# Supplementary material for: Impact of IFNL4 Genetic Variants on Sustained Virologic Response and Viremia in Hepatitis C Virus Genotype 3 Patients
Source: J Interferon Cytokine Res. 2019 Sep 27;39(10):642–9. doi: 10.1089/jir.2019.0013 (PMC6767867; doi:10.1089/jir.2019.0013)
Supplement: Supplemental data [file Supp_Fig1.pdf]

Supplementary Data

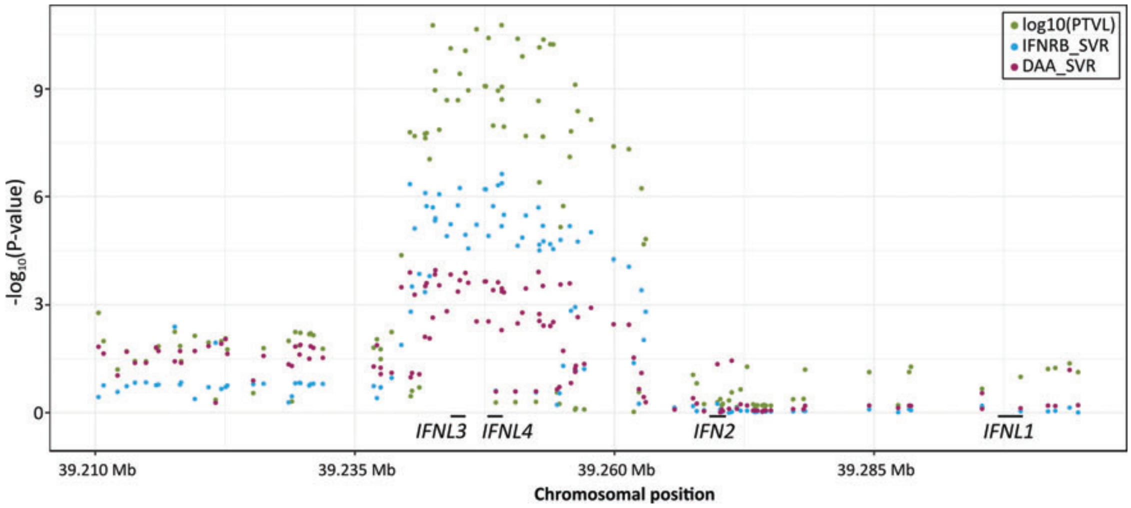

SUPPLEMENTARY FIG. S1. Regional plot of association between 114 SNPs in the *IFNL* locus and the three HCV related phenotypes [viral load $\log_{10}$(PTVL); *green dot*]; response to peg-IFN-α and Ribavirin treatment (IFNRB_SVR; *blue dot*) and to DAA treatment (DAA_SVR; *purple dot*). DAA, direct-acting antiviral; HCV, hepatitis C virus; IFNL, interferon lambda; peg-IFN-α, pegylated interferon alpha; SNP, single nucleotide polymorphism; SVR, sustained virologic response.
