# Supplementary material for: Impact of IFNL4 Genetic Variants on Sustained Virologic Response and Viremia in Hepatitis C Virus Genotype 3 Patients
Source: J Interferon Cytokine Res. 2019 Sep 27;39(10):642–9. doi: 10.1089/jir.2019.0013 (PMC6767867; doi:10.1089/jir.2019.0013)
Supplement: Supplemental data [file Supp_Table2-3.pdf]

Supplementary Table S2. Top 25 Most Significant Association Signals With Log_{10}(PTVL)

Chromosome	*SNP*	*Position*	*Beta*	P
19	rs4803221	39739129	−0.284517	1.70×10^{-11}
19	rs8105790	39732501	−0.284387	1.71×10^{-11}
19	rs8107030	39736719	−0.282882	2.21×10^{-11}
19	rs12971396	39737866	−0.278785	3.86×10^{-11}
19	rs66531907	39740675	−0.280237	4.05×10^{-11}
19	rs8099917	39743165	−0.279478	4.29×10^{-11}
19	rs7248668	39743821	−0.279242	5.77×10^{-11}
19	rs148241872	39744102	−0.279622	5.84×10^{-11}
19	rs8109889	39742770	−0.276533	7.07×10^{-11}
19	rs4803217	39734220	−0.250839	7.51×10^{-11}
19	rs28416813	39735644	−0.250354	8.74×10^{-11}
19	rs12983038	39741124	−0.274573	1.24×10^{-10}
19	rs688187	39732752	−0.241399	3.16×10^{-10}
19	rs8103142	39735106	−0.238468	3.81×10^{-10}
19	rs4803223	39746219	−0.276579	7.69×10^{-10}
19	rs73930703	39737513	−0.234871	8.34×10^{-10}
19	rs11882871	39737610	−0.234824	8.45×10^{-10}
19	rs74597329	39739155	−0.234411	8.76×10^{-10}
19	rs8105086	39732712	−0.235516	1.08×10^{-09}
19	rs201690073	39735919	−0.239132	1.10×10^{-09}
19	rs12979860	39738787	−0.233061	1.12×10^{-09}
19	rs11322783	39739153	−0.229632	1.97×10^{-09}
19	rs71169578	39733855	−0.237898	2.06×10^{-09}
19	rs11881222	39734923	−0.232485	2.07×10^{-09}
19	rs10642535	39742683	−0.232779	2.16×10^{-09}

PTVL, pretreatment viral load.

Supplementary Table S3. Top 25 Most Significant Association Signals with IFNRB_SVR

Chromosome	*SNP*	*Position*	*Odds ratio*	P
19	rs74597329	39739155	1.50464	2.35×10^{-07}
19	rs11322783	39739153	1.49242	4.26×10^{-07}
19	rs958039	39730301	1.50693	4.52×10^{-07}
19	rs12979860	39738787	1.49587	4.86×10^{-07}
19	rs8103142	39735106	1.49587	5.79×10^{-07}
19	rs73930703	39737513	1.49046	6.25×10^{-07}
19	rs11882871	39737610	1.49022	6.32×10^{-07}
19	rs12980275	39731783	1.52543	8.00×10^{-07}
19	rs581930	39733123	1.50699	8.65×10^{-07}
19	rs11881222	39734923	1.48465	1.76×10^{-06}
19	rs111531283	39738317	1.47817	1.85×10^{-06}
19	rs12982533	39731904	1.49966	1.85×10^{-06}
19	rs8105790	39732501	1.5773	2.01×10^{-06}
19	rs10642535	39742683	1.46942	2.02×10^{-06}
19	rs4803222	39739353	1.45804	3.24×10^{-06}
19	rs68018539	39741465	1.47432	3.37×10^{-06}
19	rs688187	39732752	1.4619	4.02×10^{-06}
19	rs8105086	39732712	1.45723	4.73×10^{-06}
19	rs4803217	39734220	1.44677	5.91×10^{-06}
19	rs8107030	39736719	1.53369	6.13×10^{-06}
19	rs8113007	39743103	1.45679	6.57×10^{-06}
19	rs35963157	39745695	1.45599	6.68×10^{-06}
19	rs4803221	39739129	1.53479	6.69×10^{-06}
19	rs35790907	39730755	1.45092	7.77×10^{-06}
19	rs8101517	39747741	1.44529	9.90×10^{-06}

SVR, sustained virologic response.
