# Supplementary material for: Impact of IFNL4 Genetic Variants on Sustained Virologic Response and Viremia in Hepatitis C Virus Genotype 3 Patients
Source: J Interferon Cytokine Res. 2019 Sep 27;39(10):642–9. doi: 10.1089/jir.2019.0013 (PMC6767867; doi:10.1089/jir.2019.0013)
Supplement: Supplemental data [file Supp_Table4-5.pdf]

Supplementary Table S4. Top 25 Most Significant Association Signals with DAA_SVR

Chromosome	*SNP*	*Position*	*Odds ratio*	P
19	rs688187	39732752	1.82	1.08×10^{-04}
19	rs10642535	39742683	1.77	1.22×10^{-04}
19	rs958039	39730301	1.76	1.25×10^{-04}
19	rs28416813	39735644	1.80	1.29×10^{-04}
19	rs8105086	39732712	1.81	1.43×10^{-04}
19	rs4803217	39734220	1.79	1.43×10^{-04}
19	rs8103142	39735106	1.75	2.07×10^{-04}
19	rs11882871	39737610	1.74	2.23×10^{-04}
19	rs73930703	39737513	1.74	2.24×10^{-04}
19	rs12979860	39738787	1.74	2.36×10^{-04}
19	rs201690073	39735919	1.79	2.43×10^{-04}
19	rs12982533	39731904	1.79	2.49×10^{-04}
19	rs35963157	39745695	1.68	2.53×10^{-04}
19	rs10612351	39744806	1.65	2.72×10^{-04}
19	rs581930	39733123	1.77	2.86×10^{-04}
19	rs8113007	39743103	1.75	2.97×10^{-04}
19	rs12980275	39731783	1.77	3.01×10^{-04}
19	rs955155	39729479	1.73	3.23×10^{-04}
19	rs11322783	39739153	1.69	3.50×10^{-04}
19	rs68018539	39741465	1.74	3.51×10^{-04}
19	rs111531283	39738317	1.74	3.88×10^{-04}
19	rs74597329	39739155	1.68	4.24×10^{-04}
19	rs11881222	39734923	1.74	4.28×10^{-04}
19	rs4803222	39739353	1.73	4.48×10^{-04}
19	rs35790907	39730755	1.69	5.23×10^{-04}

DAA, direct-acting antiviral.

Supplementary Table S5. Top 25 Most Significant GWAS Association Signals with Log_{10}(PTVL)

Chromosome	*SNP*	*Position*	*Beta*	P
19	rs4803221	39739129	−0.284517	1.70×10^{-11}
19	rs8105790	39732501	−0.284387	1.71×10^{-11}
19	rs8107030	39736719	−0.282882	2.21×10^{-11}
19	rs12971396	39737866	−0.278785	3.86×10^{-11}
19	rs66531907	39740675	−0.280237	4.05×10^{-11}
19	rs8099917	39743165	−0.279478	4.29×10^{-11}
19	rs7248668	39743821	−0.279242	5.77×10^{-11}
19	rs148241872	39744102	−0.279622	5.84×10^{-11}
19	rs8109889	39742770	−0.276533	7.07×10^{-11}
19	rs4803217	39734220	−0.250839	7.51×10^{-11}
19	rs28416813	39735644	−0.250354	8.74×10^{-11}
19	rs12983038	39741124	−0.274573	1.24×10^{-10}
19	rs688187	39732752	−0.241399	3.16×10^{-10}
19	rs8103142	39735106	−0.238468	3.81×10^{-10}
19	rs4803223	39746219	−0.276579	7.69×10^{-10}
19	rs73930703	39737513	−0.234871	8.34×10^{-10}
19	rs11882871	39737610	−0.234824	8.45×10^{-10}
19	rs74597329	39739155	−0.234411	8.76×10^{-10}
19	rs8105086	39732712	−0.235516	1.08×10^{-09}
19	rs201690073	39735919	−0.239132	1.10×10^{-09}
19	rs12979860	39738787	−0.233061	1.12×10^{-09}
19	rs11322783	39739153	−0.229632	1.97×10^{-09}
19	rs71169578	39733855	−0.237898	2.06×10^{-09}
19	rs11881222	39734923	−0.232485	2.07×10^{-09}
19	rs10642535	39742683	−0.232779	2.16×10^{-09}

GWAS, genome-wide association study.
