# Supplementary figures and images for: Impact of IFNL4 Genetic Variants on Sustained Virologic Response and Viremia in Hepatitis C Virus Genotype 3 Patients
Source: J Interferon Cytokine Res. 2019 Sep 27;39(10):642–9. doi: 10.1089/jir.2019.0013 (PMC6767867; doi:10.1089/jir.2019.0013)

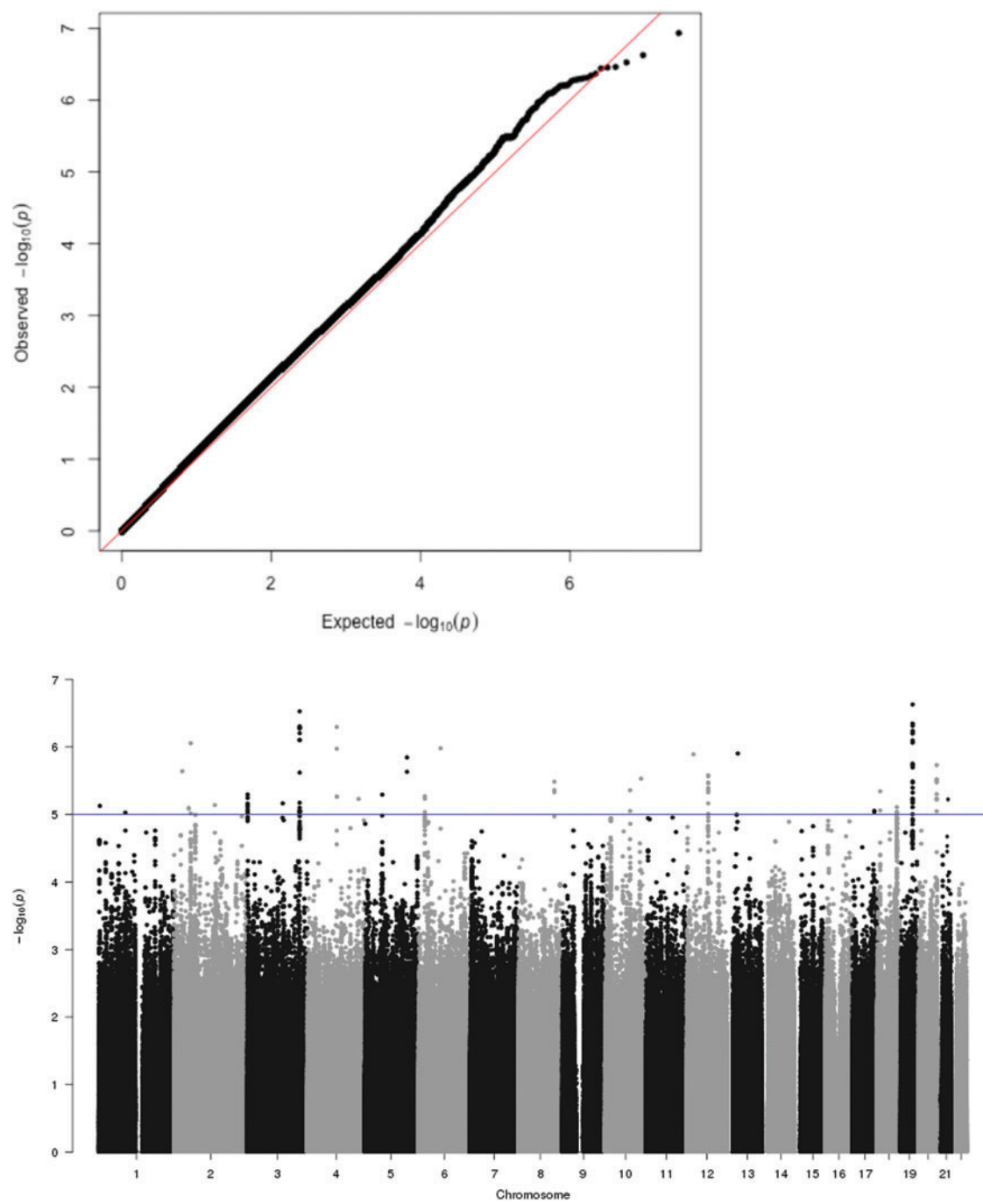

**SUPPLEMENTARY FIG. S3.** QQ plot and GWAS of IFNRB\_SVR.

Supplement: Supplemental data [file Supp_Fig3.pdf]

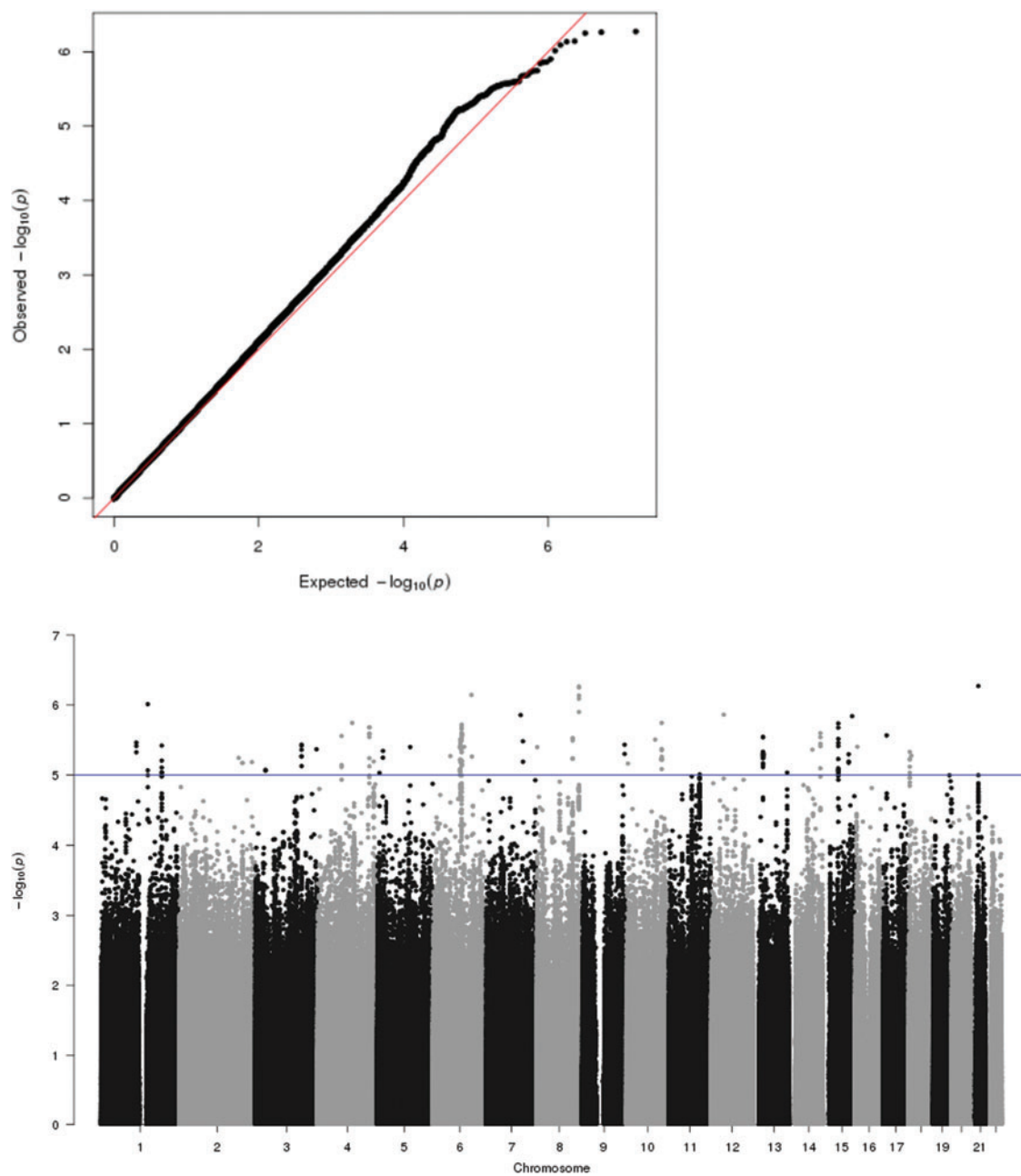

**SUPPLEMENTARY FIG. S4.** QQ plot and GWAS of DAA\_SVR.

Supplement: Supplemental data [file Supp_Fig4.pdf]
