# Supplementary material for: Impact of IFNL4 Genetic Variants on Sustained Virologic Response and Viremia in Hepatitis C Virus Genotype 3 Patients
Source: J Interferon Cytokine Res. 2019 Sep 27;39(10):642–9. doi: 10.1089/jir.2019.0013 (PMC6767867; doi:10.1089/jir.2019.0013)
Supplement: Supplemental data [file Supp_Table6-7.pdf]

Supplementary Table S6. Top 25 Most Significant GWAS Association Signals with IFNRB_SVR

Chromosome	*SNP*	*Position*	*Odds ratio*	P
19	rs74597329	39739155	1.59	2.35×10^{-07}
3	rs6445096	172780967	1.57	2.97×10^{-07}
19	rs958039	39730301	1.58	4.52×10^{-07}
19	rs12979860	39738787	1.57	4.86×10^{-07}
3	rs6445097	172781729	1.56	4.95×10^{-07}
4	rs76755915	97959607	0.22	5.06×10^{-07}
3	rs9833380	172780363	1.56	5.16×10^{-07}
3	rs12635576	172777249	1.56	5.22×10^{-07}
3	rs2861067	172783277	1.56	5.35×10^{-07}
19	rs8103142	39735106	1.56	5.79×10^{-07}
3	rs1113587	172783511	1.56	6.25×10^{-07}
19	rs73930703	39737513	1.56	6.25×10^{-07}
19	rs11882871	39737610	1.56	6.32×10^{-07}
3	rs6781476	172774448	1.54	7.88×10^{-07}
3	rs7645546	172782231	1.55	7.94×10^{-07}
19	rs12980275	39731783	1.55	8.00×10^{-07}
19	rs581930	39733123	1.56	8.65×10^{-07}
2	rs13401937	55252214	1.94	8.81×10^{-07}
6	rs2492965	73155175	0.35	1.05×10^{-06}
4	rs78736235	97948861	0.22	1.06×10^{-06}
13	rs7332270	35431953	0.61	1.25×10^{-06}
12	rs75707332	22174937	2.56	1.28×10^{-06}
5	rs72799981	142530386	6.64	1.43×10^{-06}
19	rs11881222	39734923	1.54	1.76×10^{-06}
19	rs111531283	39738317	1.54	1.85×10^{-06}

Supplementary Table S7. Top 25 Most Significant GWAS Association Signals with DAA_SVR

Chromosome	*SNP*	*Position*	*Odds ratio*	P
21	rs35384524	22389690	0.28	5.35×10^{-07}
8	rs59888390	136001312	0.26	5.45×10^{-07}
8	rs66485405	136002315	0.26	5.63×10^{-07}
6	rs56378326	122991423	0.00	7.19×10^{-07}
8	rs6578047	136009494	0.26	7.33×10^{-07}
8	rs7833239	135981618	0.26	8.11×10^{-07}
1	rs116399066	149217804	6.39	9.70×10^{-07}
8	rs72724379	135989141	0.27	1.25×10^{-06}
7	rs76810409	109096452	0.00	1.39×10^{-06}
15	rs62042908	93849243	2.56	1.44×10^{-06}
4	rs7670514	111133368	2.40	1.80×10^{-06}
15	rs12323963	49231111	2.75	1.83×10^{-06}
6	rs57030374	91840403	3.72	1.90×10^{-06}
6	rs9362832	91840004	3.71	2.05×10^{-06}
4	rs10023300	165766741	2.43	2.08×10^{-06}
4	rs10011468	165766739	2.43	2.08×10^{-06}
15	rs60658076	49228615	2.70	2.10×10^{-06}
6	rs9362827	91835828	3.67	2.20×10^{-06}
6	rs9362833	91840612	3.62	2.50×10^{-06}
6	rs9359924	91839956	3.62	2.53×10^{-06}
4	rs13131247	165767001	2.39	2.54×10^{-06}
6	rs9359923	91839622	3.61	2.55×10^{-06}
6	rs9353796	91840664	3.62	2.60×10^{-06}
4	rs4337691	165761928	2.51	2.60×10^{-06}
6	rs6936521	91838828	3.59	2.65×10^{-06}
